# Effective dean vortex separation at reduced flow rates towards rare cell sorting

**DOI:** 10.1038/s41598-026-40845-4

**Published:** 2026-02-25

**Authors:** Emma Dupont, Lionel Artinyan, Céline Brunin, Marie Piecyk, Léa Payen, Emmanuelle Laurenceau, Gilles Simon, Jérôme Degouttes, Damien Le Roy, Anne-Laure Deman

**Affiliations:** 1 Université Claude Bernard Lyon 1, CNRS, INSA Lyon, Ecole Centrale de Lyon, CPE Lyon, INL, UMR5270, Villeurbanne, 69621 France; 2 Université Claude Bernard Lyon 1, CNRS, Institut Lumière Matière, UMR5306, Villeurbanne, 69100 France; 3 Laboratoire de Biochimie Et Biologie Moléculaire, Groupe Hospitalier Sud, Hospices Civils de Lyon, Pierre Bénite, 69495 France

**Keywords:** Engineering, Mathematics and computing, Physics

## Abstract

The study focuses on the design of a spiral microfluidic device, aiming to efficiently sort particle by size, at tailored flow rate for downstream processing. While spiral devices exploiting Dean vortices are recognized for their high-throughput capabilities, they often require high flow rates that limit their integration with other microfluidic functions and reduce sorting performance. Our work aims to design a low flowrate-operating-spiral (~ 50 mL/h) by investigating the influence of spiral geometric parameters and flow conditions on sorting efficiency. Through combined experimental and theoretical analysis, we evaluate how particle size and flow dynamics determine particle positions within the spiral, validating underlying models. This approach provides valuable insights for optimizing spiral microfluidic systems particularly their design, performance, and versatility in applications such as the isolation of rare cell isolation. The spiral design achieved efficient size-based sorting of 10 and 15 μm microbeads at a flow rate of 50 mL/h. When applied to biological samples, the system removed 89% of white blood cells from a 1:1 lysed blood sample (≈ 10⁷ cells/min) while maintaining a recovery of more than 75% for all tested CTC-mimicking cells.

**Supplementary Information:**

The online version contains supplementary material available at 10.1038/s41598-026-40845-4.

## Introduction

Microfluidics has been attracting growing attention in recent years as it enables the miniaturization of analyses and the repeated execution of multiple assays, with direct applications in personalized medicine. Beyond its versatility, microfluidic platforms allow highly automated and real-time control of experimental conditions, valuable for manipulating individual biological objects such as cells, including the isolation of rare cell populations for early cancer diagnosis from liquid biopsies^1,2^. Cell sorting in microfluidics typically relies on two strategies: (i) biological discrimination based on surface markers, offering high specificity^3^, and (ii) physical discrimination based on intrinsic properties such as size, deformability or dielectric characteristics^4^. These physical differences can be exploited using a wide range of manipulation techniques^5^, such as acoustic or electrical methods^6,7^, filtration^8^, or hydrodynamic methods^9^, which are generally less specific but enable faster sorting. These two approaches are complementary, and implementing them in cascade may be particularly relevant when dealing with rare cells^10–12^. Within the latter strategy, passive hydrodynamic methods, and in particular spiral microchannels exploiting Dean vortices, have emerged as powerful platforms for high-throughput sorting. However, as with all passive hydrodynamic approaches, their performance critically depends on precise control of channel geometry. Furthermore, their ability to operate at high flowrates can be considered as an advantage, as it reduces the processing time, but also as a disadvantage, particularly in complex workflows involving several microfluidic functions, each operating at a specific flow rate range.

In straight rectangular channels, particles transported in laminar flow are subjected to drag forces along the flow direction ($\:{F}_{Drag}$), while across the cross-section, they experience wall-induced and shear-gradient lift forces, which lead to an overall inertial lift force $\:{F}_{L}$^4^:

1 $$\:{F}_{L}=\frac{\rho\:{U}^{2}{a}^{4}}{{{D}_{\mathrm{h}}}^{2}}{f}_{L}\left(Re,{x}_{p}\right)\:\left(N\right)$$

where $\:\rho\:$ represents the fluid density, U whether the mean^13^ or the maximum^14^ velocity of the fluid (mean velocity will be used in this study), $\:{D}_{\mathrm{h}}=\frac{2wh}{w+h}$ the hydraulic diameter of the channel, $\:a$ the particle diameter and $\:{f}_{L}$ is the lift coefficient, which depends on the $\:Re$, the Reynolds number, and $\:{x}_{p}$, the position of the particle in the channel. This inertial lift force leads particles to migrate toward equilibrium positions that strongly depend on their size^15^. These inertial effects already provide a route to size-based separation.

Curved channels introduce an additional level of control. The bending of streamlines generates secondary flows, known as Dean vortices. Their intensity is captured by the dimensionless Dean number (De), initially introduced as K by Dean^16^ and later revised in subsequent studies, as noted by Saffar et al.^17^. The most commonly used expression for Dean’s number is the one given by Berger et al.^18^:

2 $$\:\:De={Re}\sqrt{\frac{{D}_{\mathrm{h}}}{2R}}\:$$

where $\:R$ is the radius of curvature of the channel.

These vortices superimpose a transverse drag force on particles, altering their equilibrium positions:

3 $$\:{F}_{\mathrm{D}}=\rho\:\frac{{U}^{2}a{{D}_{\mathrm{h}}}^{2}}{R}\:\left(\mathrm{N}\right)\:$$

Interestingly, the Dean drag force scales linearly with particle size ($\:{F}_{\mathrm{D}}\sim\:\:a$​) whereas the inertial lift force scales with the particle size at the fourth power ($\:{F}_{\mathrm{L}}\sim\:{a}^{4}$​). This contrast amplifies size-dependent migration: small particles are carried away by vortices across the channel, while larger ones stabilize near the inner wall where lift and Dean forces balance^19,20^. This interplay underpins the use of spiral channels for the separation of particles or cells. In order to determine whether or not a population of particles is entrained in the vortices, predictive criteria are used, such as the confinement ratio $\:(\frac{a}{{D}_{\mathrm{h}}}>0.07)$^21,22^ and the ratio of the lift force-to-Dean force ratio $\:{(R}_{\mathrm{f}}\sim\frac{{\mathrm{F}}_{\mathrm{L}}}{{\mathrm{F}}_{\mathrm{D}}}>0.08)\:$[24]. Nevertheless, these criteria are largely empirical^13,23,24^, and only validated for limited geometric parameters.

Leveraging these principles, Dean vortices have been exploited for size-based separation of mixed populations.

Two scenarios are typically distinguished. In the first one, only the larger population satisfies the focusing condition, while smaller particles are carried by the vortices across the cross-section. Separation can then be enhanced by channel length optimization or trapezoidal cross-sections that displace vortices outward^25–28^. Among these approaches, Shen et al. developed spiral microchannels incorporating micro-obstacles, which locally reduce the channel width and thereby increase the Dean number and the strength of the Dean vortices^29^. Using diluted blood samples (2.5% hematocrit) spiked with rare tumor cells (10¹–10⁴ cells/mL), they achieved tumor cell recovery rates exceeding 95% at flow rates between 180 and 360 mL/h. In addition, Jeon et al. sought to enhance particle sorting efficiency by developing a multi-dimensional double spiral (MDDS) design, which increases the effective length of the inertial microchannel^30^. This spiral configuration has been widely employed in various bioengineering applications, among them, it has been used to purify T-cell populations by removing up to 85% of unactivated T cells while recovering approximately 80% of activated T cells at a flow rate of 210 mL/h^31^.

**Fig. 1:**
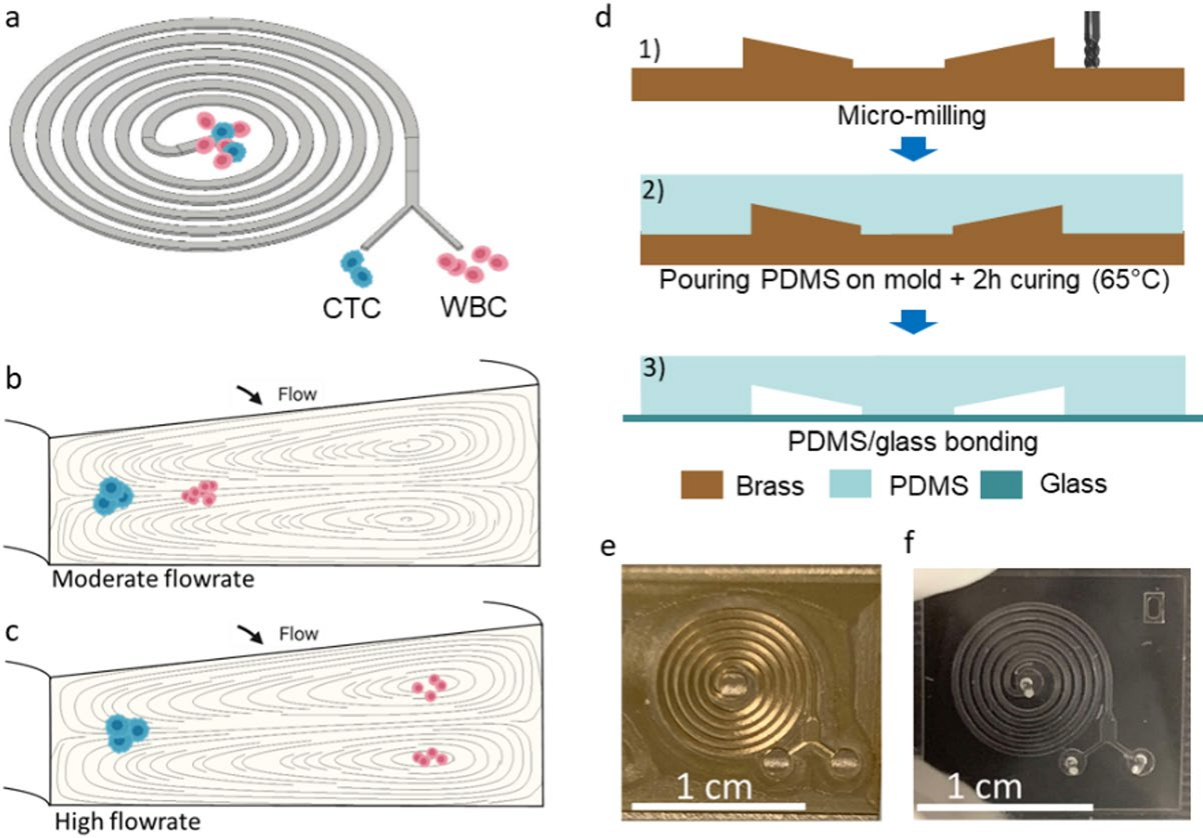
(**a**) Schematic illustration of the separation principle based on Dean vortices in a spiral microfluidic channel. The sample is introduced through the central inlet, and sorted cells are collected at the outlet. (**b**,**c**) Schematic cross-sectional views of the spiral channel at the outlet: (**b**) at moderate flow rates, both particle populations focus near the inner channel wall. (**c**) At higher flow rates, larger particles remain focused near the inner wall, while smaller particles are carried toward the outer wall, near the vortex center. (d) Chip fabrication process of the microfluidic chip. (**e**) Picture of the brass spiral mold. (**f**) Picture of the final microfluidic chip in PDMS.

In the second scenario, both populations are focused at distinct positions (Fig. 1.b), enabling separation, provided their respective streams are sufficiently spaced apart. By adjusting both the channel design and the flow rate, particle positions can be selectively controlled through the Dean number (De)^13,17,32^. As the flow rate, and therefore De, increases, particles leave their equilibrium position and migrate towards the outer wall^19,33,34^. The flow rate at which this cross-sectional migration occurs depends on particle size^35^. A practical size-based separation strategy therefore consists in operating at a flow rate at which only one of the two populations has already shifted (Fig. 1.c). Although the behavior of the particles within Dean vortices or in the equilibrium position near the inner wall are well established, the mechanism associated with the shift of the equilibrium position from the inner to the outer wall remains a subject of debate, with hypotheses ranging from force balance variations^33^ to migration into vortex centers at high flow rates^28,34^.

Overall, because understanding of the mechanisms involved has been determined empirically and some remain poorly understood, Dean vortices remain challenging to implement. Geometric parameters such as height, width, aspect ratio, curvature of the channel, number of turns, or cross-sectional shape, all contribute, often in an interdependent manner, making comparisons between studies difficult^36^.

As a general guideline, to separate particles around ten microns in size (comparable to cells), spiral channels are typically designed with widths ranging from 100 μm to 500 μm. Thus, despite numerous empirical guidelines^17,37^, systematic design rules remain limited.

Another main limitation of the Dean vortex is that, as particle behavior in Dean spiral devices is strongly dependent on flow velocity, most systems reported in the literature operate at high flow rates (typically above 100–4000mL/h). Beyond inducing higher shear stress for the cells, such high-throughput operation limits compatibility with other on-chip functions. Indeed, with the increasing demand for highly precise separation in biological sample sorting, many microfluidic solutions (such as DLD or DEP-based sorting^38,39^ have been developed to operate at low flow rates, enabling improved control over particle trajectories and enhanced selectivity. Although spiral microfluidics has demonstrated strong sorting capabilities, the restricted range of operating regimes constrains the versatility of the Dean vortex approach. Operating at lower flow rates could facilitate the integration of more complex workflows, including pre-enrichment functions.

In this work, we investigate Dean vortex–based cell sorting at flow rates significantly lower (~ 50 mL/h), than those typically used in inertial microfluidics. Reducing operating flow velocity involves reducing the cross-sectional dimension of the channel and imposes some constraints on the channel geometry. We experimentally studied the migration of suspended targets from the inner wall to the outer wall of curved microchannels, as well as their resulting equilibrium positions. The experimental observations were complemented by finite-element simulations of the fluid flow in the corresponding geometries, enabling us to characterize the hydrodynamic conditions underlying the observed separation performance. Using model microbeads, we then established relationships between the geometric parameters of the channel, the applied flow rates, and the separation performances obtained in the targeted flow rate ranges. We then implemented the most effective system using cancer cell lines and white blood cells to address its potential in cell isolation applications for liquid biopsy, such as sorting circulating tumour cells from blood samples or tumour cells released into other body fluids as urine or intraperitoneal fluid.

## Materials and methods

### Microfluidic chip fabrication

Devices consist of microchannels made up of 6-loop spirals whose various dimensions, such as channel width and height and aspect ratio, vary from one to another. The master molds were fabricated by micro-milling (CNC Mini-Mill/3, Minitech Machinery Corporation) on brass (Fig. 1.d)^40^. The trapezoidal cross-section required a two-step milling process: first, the horizontal contour of the spiral with 500 μm and 200 μm flat-end mills; then, the inclined top wall with a 200 μm ball-end mill using 1 μm z-steps. The spiral design features an inlet located at its center and an outlet positioned at the exterior, which divides into two separate branches.

The microchannels were then fabricated by casting PDMS (Sylgard Silicone Elastomer, 10:1 base and curing agent mixing ratio) on the mold and curing it at 65 °C during 2 h. After curing, the PDMS was peeled from the mold and plasma bonded to a 1 mm thick flat glass slide to complete the microchannel. Input and output ports of 0.5 mm were punched prior to bonding step with the Uni-Core TM Puncher (Fig. 1.e).

### Target particles: beads and cells

The test samples consist of three batches of particle-containing liquid suspensions: (i) a suspension of polystyrene beads, (ii) pre-treated blood samples, (iii) a suspension of tumor cells derived from cell lines.

For bead-based experiments, fluorescent polystyrene microspheres (ThermoFisher Scientific) of 10 μm diameter (9.9 ± 0.09 μm, blue) and 15 μm (15.4 ± 0.139 μm, red) were suspended in phosphate-buffered saline (PBS). A mixed solution was prepared with both bead types at equal concentrations of 1 × 10^6^ beads/mL.

White blood cells (WBCs) were obtained from blood samples collected in K2-EDTA tubes (BD Vacutainer) from healthy volunteers. According to the Declaration of Helsinki ethical guidelines, each participant was informed and signed a consent form as part of the CIRCAN MAP protocol (ID-RCB: 2021-A02212-39) approved by the *Comité de Protection des Personnes* Sud Est I on December 13th 2021. Red blood cells (RBCs) were removed by adding a 1x lysis buffer (EurX^®^, E0326-02) at a 1:4 (v/v) blood-to-buffer ratio for 10 min at room temperature (RT), followed by centrifugation at 500 × g for 10 min. The supernatant, containing lysed RBCs, was discarded and the WBCs were collected from the pellet at mean concentration of 9 × 10⁶ cells/mL.

Tumor cell lines MDA-MB-231, HCC827, PC3, and MCF7 were cultured under standard conditions. For each of these cell lines, samples containing 3 × 10⁶ to 3 × 10⁷ cells/mL were prepared. Although this CTC concentration is higher than the one typically found in patient blood, it was necessary in order to study their behavior under fluorescence.

Both WBCs and tumor cells were stained with Hoechst dye (1 µL/mL, blue) for 15 min at 37 °C, followed by three centrifugation (1000 rpm, 5 min) and resuspension steps in PBS. Each cell population’s behavior was then independently characterized.

### Acquisition method

The various sample solutions were introduced into the spiral microchannels using a syringe pump (DKInfusetek, ISPLab02). Fluorescence imaging was performed using a Thunder microscope (Leica Microsystems) with exposure times of 50 ms for bright-field and 500 ms for fluorescence images. Fluorescent signal intensities corresponding to each bead population were extracted using ImageJ software.

For the boxplot representations, the central line represents the median fluorescence intensity across the channel width, while the lower and upper edges of the box correspond to the 25th and 75th percentiles, respectively. The whiskers extend to the most extreme values that are not considered outliers.

The mean cell diameter was calculated based on optical microscopy images obtained at 63× magnification (Leica Microsystems), with measurements performed on a minimum of 100 individual cells.

### Numerical simulations

The laminar flow study in a spiral channel was numerically performed using COMSOL Multiphysics, version 6.3. The CAD design files used for COMSOL simulations are based on measurements of the fabricated brass molds. The stationary (steady-state) study was conducted by applying an inlet flow velocity corresponding to flow rates between 20 and 230 mL/h, while a zero-pressure condition was set as the outlet boundary condition. A no-slip boundary condition was applied to the channel walls (a zero-fluid velocity at the wall). The fluid properties were the ones of water at ambient temperature (density: 1000 kg/m^2^; kinematic viscosity: 10^−6^ m^2^/s).

The Navier–Stokes equations were solved using the finite element method (FEM) solver, assuming a three-dimensional, steady-state, laminar flow. The governing equations are:

$$\rho (u \cdot \nabla )u = \nabla \cdot \left[ { - pI + \mu \left( {\nabla u + \left( {\nabla u} \right)^{T} } \right)} \right] + F\;and\;\nabla \cdot u = 0,$$

where $\:u$ is the velocity field, $\:p$ is the fluid pressure, $\:\rho\:$ is the fluid density, µ is the dynamic viscosity, and $\:F$ is a constant. A tetrahedral mesh was used for the 3D model, consisting of volume elements of approximately $\:{6.10}^{3}\:{\mu\:\mathrm{m}}^{3}$.

The positions of the vortex centers, obtained in simulation, were determined by locating the centers of the streamlines of the velocity field in cross-sectional planes perpendicular to the channel direction at the outlet of the spiral, after six turns. The position was determined with an uncertainty of 3 μm, corresponding to half the width of the smallest streamline ellipse. Regarding the center position analysis, the average x-position between the centers of the upper and lower vortices was considered.

## Results and discussion

### Geometric characteristics of the spiral devices

Spiral devices designed aim to operate at moderate flow rates to ensure compatibility with efficient low-flow-rate functions and integrated workflows. The restriction to sorting flow rates of around 50 mL/h imposes a geometric constraint on the designed spiral channels. Their cross-sectional area was then reduced (~ 0.01 mm^2^) compared with the typical dimensions reported in the literature (~ 0.05 mm^2^). We fabricated devices with varying geometries, all featuring a trapezoidal cross-section and six turns.

Eight devices (S0 to S7) with different geometries were built, varying in channel width, upper wall slope and aspect ratio. The first separation strategy, presented in the introduction, in which the large population is focused while the small one is dragged into the vortices, was evaluated with device S0 (see Supplementary Table 1). However, it quickly showed its limitations: the large beam spread of the small microparticles impedes their separation from the large particles in narrow channels (see Supplementary Fig. 1). We therefore adopted the second separation strategy, in which one population concentrates near the inner wall and the other stabilizes near the outer wall, for devices S1 to S7. They were therefore all designed so that the two types of particles studied met the focusing criteria (see Supplementary Table 2). Their characteristics are listed in Table 1. The aspect ratio is defined as h/w, the smaller it is, the more flattened the channel is. Hydraulic diameters $\:\left({D}_{h}\right)$ were determined using the internal height of the channel, as suggested by Wu et al.^28^. The slope of the upper wall corresponds to the ratio of the height difference between the inner and outer walls, *∆h*, and the channel width, *w (*$\:s\:\left(\%\right)=\:\varDelta\:h/\omega\:$). For all devices, $\:De$ was calculated at a representative $\:Re$ of 75 (targeted flow rate range). At this value, for all devices, De is ranging between 7,5 and 10,2 μm.

**Table 1:** Dimensions and flow-related characteristics for devices S0–S9. A visual representation is presented in Supplementary Fig. 2. Method: E/S = Experimental and simulation, S = Simulation. Varied parameter: s = Slope, w = width, AR = Aspect ratio.

Spiral number		S0	S1	S2	S3	S4	S5	S6	S7	S8	S9
Method		E/S	E/S	E/S	E/S	E/S	E/S	E/S	E/S	S	S
Varied parameter		$\:a/{D}_{\mathrm{h}}$	Slope/ Width	Slope/ AR	Slope	Width	Width	AR	AR	Slope	AR
Width (µm)		335	250	250	250	200	350	260	335	250	250
Mean height (µm)		128	73	74	69	75	73	50	90	85	100
Inner height (µm)		105	60	68	50	65	60	45	65	85	95
Outer height (µm)		150	85	79	88	85	85	55	115	85	105
Slope$\:\:({\varDelta\:}{h}/{\omega\:})\:$(%)		13	10	4	15	10	7	4	15	0	4
Aspect ratio$\:\:({h}^{w})$		0.38	0.29	0.29	0.28	0.38	0.21	0.19	0.27	0.34	0.40
$\:{{D}}_{{h}}$ (µm)		160	97	107	83	98	102	77	109	127	138
$\:{R}$ (outlet) (mm)		4.7	3.8	3.8	3.8	3.2	4.9	3.9	4.7	3.8	3.8
Re = 75	De (µm)	9.8	8.5	9.0	7.9	9.3	7.7	7.5	8.1	9.8	10.2
	U (m/s)	0.47	0.78	0.70	0.90	0.76	0.73	0.98	0.69	0.59	0.54
	Flowrate (mL/h)	72	51	46	56	41	67	46	75	45	49

We further simulated the vortices generated in these devices for different flow rates, as well as in two additional designs (S8 and S9) that were not fabricated. We first investigated the vortex center positions, before comparing them with the equilibrium positions reached by particles near the outer wall at high flow rates. We then compared the separation performances of 10 and 15 μm diameter fluorescent microparticles in the different devices, and finally implemented the best-performing geometry with different cell populations, which are intrinsically more deformable and polydisperse, cancer cell lines and white blood cells.

### Inner wall to outer wall shift of focused particles

We investigated the displacement of the microparticles stream along the device S1, as a function of the flow rate. A top-view image was taken at the spiral outlet, capturing the position of two fluorescent bead populations: 10 μm beads (blue) and 15 μm beads (red). These images were acquired at various flow rates ranging from 10 to 120 mL/h (Fig. 1.a). Both bead populations effectively focus into a single streamline, as expected for these channel dimensions. The lateral position of the particles across the channel width changes with the flow rate, in agreement with previous studies^19,34^.

**Fig. 2:**
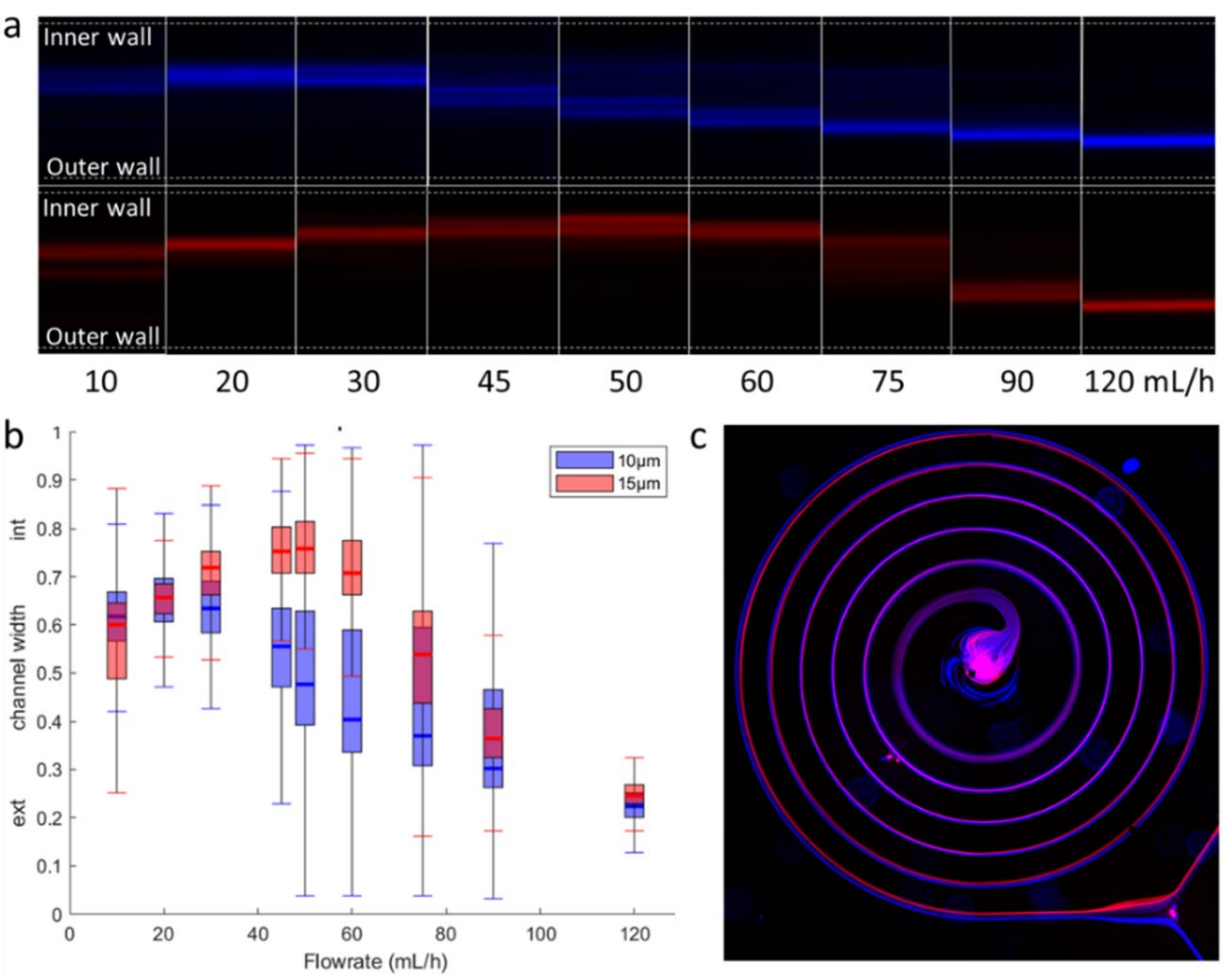
(**a**) Fluorescence signal of circulating 10 μm (top) and 15 μm (bottom) beads at the outlet section of the S1 spiral. (**b**) Fluorescence intensity position of 10 μm and 15 μm beads in normalized the channel width at the spiral outlet in S1. (**c**) Superimposed fluorescence image of 10 and 15 μm diameter beads (in blue and red respectively) along the entire spiral.

As shown in Fig. 2.a, both particle populations focus near the inner wall at low flow rates. As the flow rate increases, the focused stream gradually shifts toward the outer wall (see Supplementary video 1). This transition occurs at distinct thresholds: 30 mL/h for 10 μm beads, and 60 mL/h for 15 μm beads. Such size-dependent lateral displacement of particle beams enables separation at intermediate flow rates, where large and small particles occupy opposite sides of the channel. Fluorescence intensity profiles across the normalized channel width, shown in Fig. 2.b, confirm that at 50–60 mL/h, larger 15 μm beads remain near the inner wall while smaller 10 μm beads localize near the outer wall. The spatial separation progressively develops along the spiral (Fig. 2.*c*) and is visible at the exit, where the two populations exit through different paths.

A similar inner-to-outer-wall shift of particles was observed in all other tested devices (S1 to S7 in Table 1), though the transition flow rate varied with the channel geometry. We further investigated whether the displacement of particles towards the outer wall reflects particle migration towards the centers of high-speed vortices, and how the spiral design parameters modulate this behavior.

### Modeling fluid flow in devices

#### Location of the vortices.

**Fig. 3:**
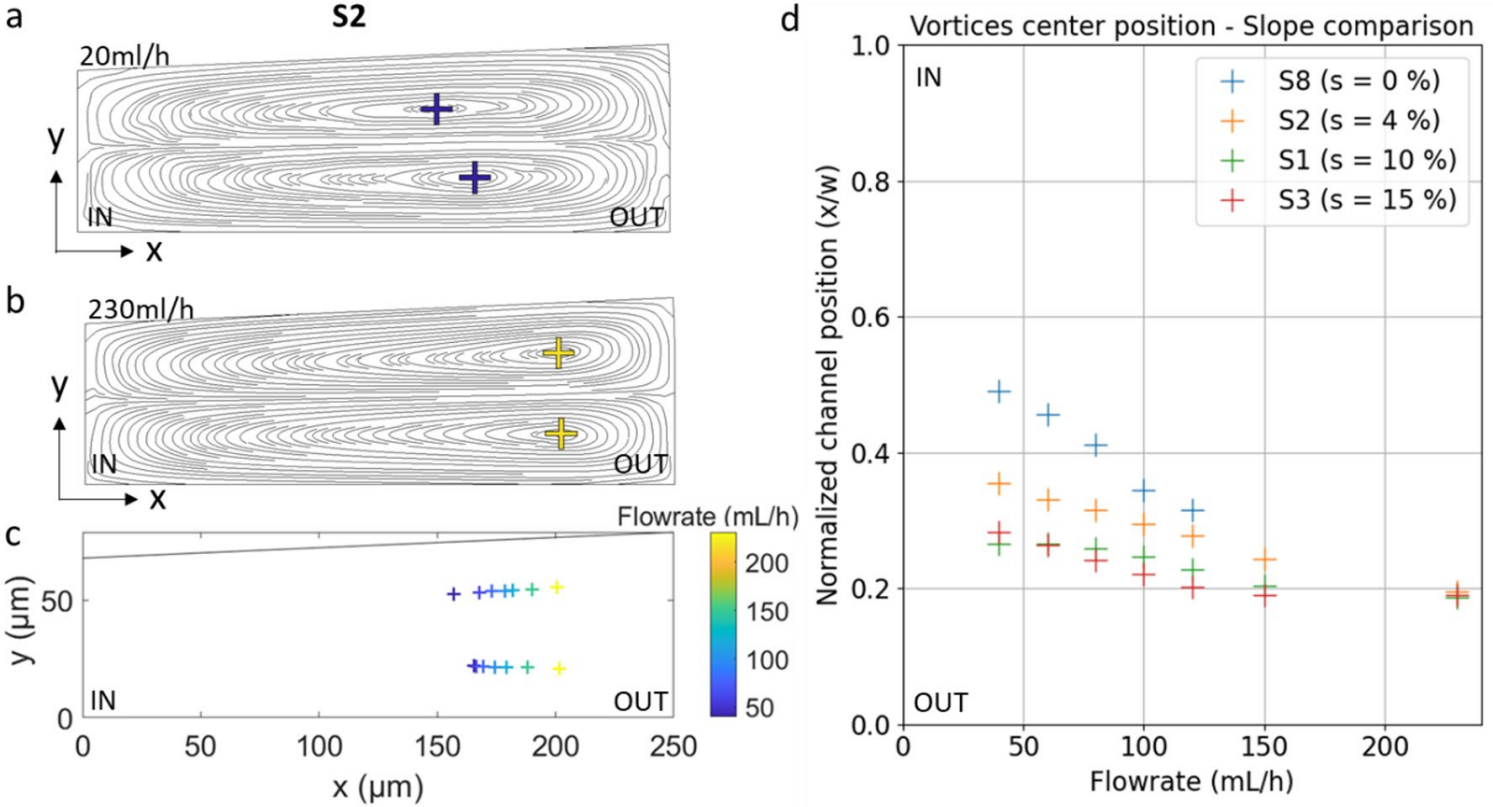
(**a**,**b**) Velocity field-based streamlines in the channel cross-section at the outlet of the S2 spiral at 20mL/h (a) and 120mL/h (b). Cross represents the vortices centers. x and y represent respectively the width and height of the channel section. (**c**) Vortices center positions in S2 cross section depending on the flowrate. (**d**) Lateral position of vortex centers across the normalized channel width for varying flow rates (40 to 230 mL/h). Effect of parameters of interest on vortex center location channel slope in S8, S2, S1 and S3.

We implemented the channel designs S1 to S9 in COMSOL to analyze fluidic dynamics at the spiral outlet, focusing on the position of the vortex centers. Their location was obtained from the centers of the streamlines of the velocity field in cross-sectional planes perpendicular to the channel direction (Fig. 3a,b).

At the outlet cross section, increasing the flow rate leads to the formation of two counter-rotating Dean vortices, located in the upper and lower halves of the channel. However, below a certain De threshold (which depends on the device geometry), vortex formation remains incomplete, and the flow exhibits multiple unstable and disorganized vortices^17,41,42^. In our experiments, most devices exhibit the formation of two stable vortices from around 20 mL/h, while devices S3 and S6 require higher flow rates, up to 40 mL/h, for well-defined vortices to appear. Regardless of the geometry, for each device, as the flow rate increases, the vortex centers progressively shift closer to the outer wall of the channel. This behavior is particularly significant in S2, with a maximum displacement of 40 μm between the inner and the outer position (Fig. 3c). This outward migration can be explained by the combined increase of the centrifugal force and the fluid inertia with the flow rate. In this case, the corresponding De rises from 3.9 to 44.4, for 20 mL/h and 230 mL/h respectively, considering a curvature radius of 3.75 mm at the spiral outlet. In parallel, the radius of curvature also significantly influences the vortex position. Simulations show that, for all tested devices, the centers of the vortices are located farther outward after the first turn of the spiral (*R* = 1.2–1.35 mm) compared to their positions after six turns (*R* = 3.75–4.75 mm). As the radius of curvature decreases, the Dean number increases, resulting in an outward shift of the vortices (see Supplementary Fig. 3), consistent with the displacement observed when the flow rate, and thus De, increases.

The effects of three geometric parameter, i.e., channel width, aspect ratio and slope, on the displacement of vortex centers between low (20 mL/h) and high (230 mL/h) flow rates were analyzed. Regarding the influence of channel width (200 to 350 μm) on vortex displacement (see Supplementary Fig. 4), it is difficult to establish a general trend, but it should be noted that the narrowest channel (200 μm) has a much more centered vortex position at low flow rates than the wider channels. With regard to the aspect ratio (0.2 to 0.4), a lower aspect ratio tends to displace the vortices outward, regardless of the flow rate applied. The influence of the channel slope was also investigated. Previous studies generally report slopes between 6% and 8%, typically associated with larger channel widths (w ≈ 500 μm)^43^. Figure 3.d shows normalized lateral position of vortex centers across the channel width for varying flow rates for devices S8, S2, S1, and S3, which have similar aspect ratios (0.29 to 0.34) and widths (250 μm), but respective slopes of 0%, 4.4%, 10.0%, and 15.2%. At low flow rates, vortices in rectangular channels ($\:s=0\%$ ) are located at the center of the channel width, whereas vortices of channels with an inclined upper wall on the outside, are located outwards. The steeper the slope, the further outward the vortices are positioned. At higher flow rates, vortices in all devices progressively move outward, converging to a similar final position, approximately 20% from the outer wall of the channel.

An increase in the Dean number, (high flow rate or smaller curvature radius), drives the vortex centers toward the outer channel wall. With regard to the geometry of the channel (in the ranges considered in this study), the smaller the aspect ratio, the further the vortex centers are positioned outside the channel. Furthermore, the slope of the channel has a significant impact on Dean flow, shifting the vortices outward as the slope increases. In the next section, we examine how, in turn, these vortex positions directly govern particle focusing and equilibrium locations.

#### Focusing mechanism of beads at high flow rates

**Fig. 4:**
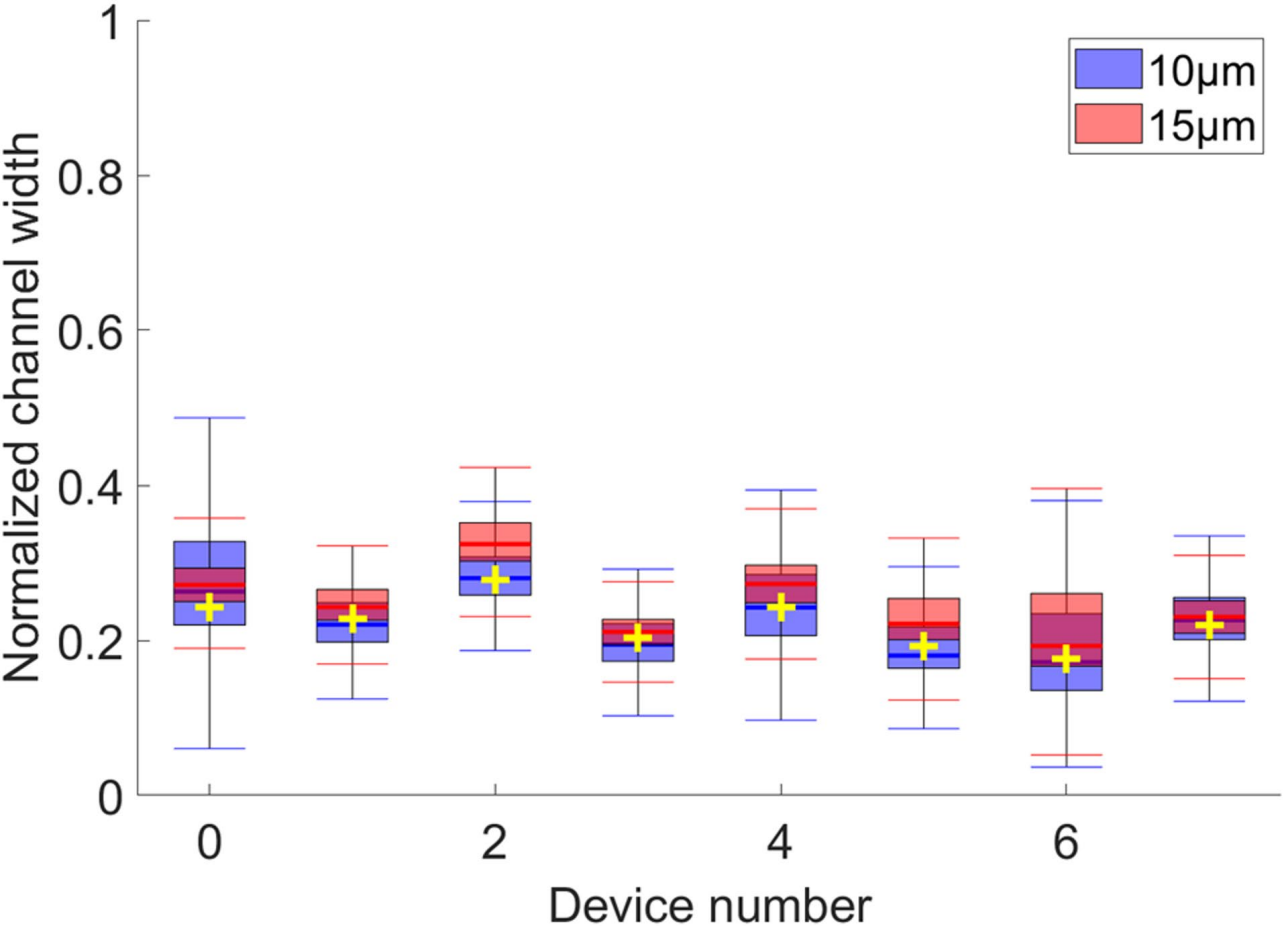
Boxplots showing the distribution of fluorescence intensity across the normalized channel width for 10 μm (blue) and 15 μm (red) microbeads in devices S1–S7. Data were collected at the highest flow rate tested for each device: 120 mL/h (S0–S4, S7), 150 mL/h (S5), and 230 mL/h (S6). Vortex centers at these flow rates are indicated by yellow crosses.

If the equilibrium position near the inner wall, due to a balance of forces between the Dean force and the inertial lift force, is well argued in the literature, the outer equilibrium position is less discussed. As explained earlier, Guan et al.^34^ hypothesized that particles find an equilibrium position in the center of vortices at high flowrates. We have therefore compared the beads equilibrium positions, obtained experimentally at high flow rates for devices S1 to S7, with the position of the center of the vortices, obtained by simulation for these same devices. Figure 4 shows the location of 10 μm and 15 μm particles at high flow rates in the channel width, the cross representing the positions of the vortex centers, for the 7 studied devices. Beyond a certain flow rate, after their transition from the inner to the outer wall of the channel, the beads concentrate into a narrow beam, located outside the channel. This flow rate is reached for most of the devices at 120 mL/h. For devices S5 and S6, the 120 mL/h flow rate was not sufficient for the particles to achieve their displacement, so the data for S5 and S6 were acquired and calculated at 150 mL/h and 230 mL/h, respectively. They both exhibit a smaller aspect ratio (~ 0.2), and such geometry favors particles remaining at their inner equilibrium position even at higher flow rates (see Supplementary Fig. 5)^44^.

Overall, the beads consistently focused into narrow streams aligned with the simulated vortex positions, regardless of their size. These findings support the hypothesis that particles tend to migrate toward the vortex centers at high flowrates.

### Particles separation depending on channel parameters

**Fig. 5:**
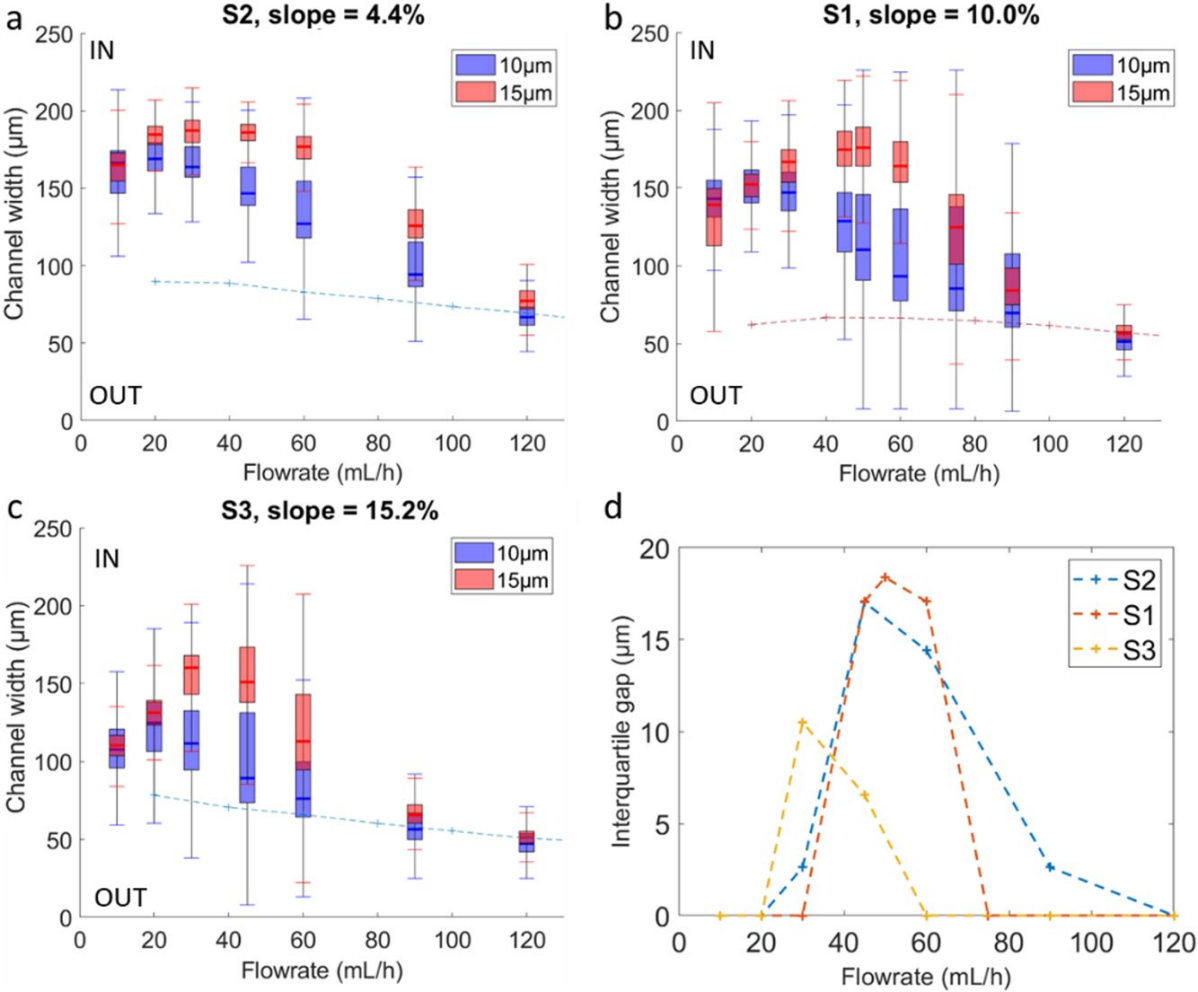
Fluorescence intensity position of 10 μm and 15 μm beads in normalized the channel width at the spiral outlet in (**a**) S2, (**b**) S1 and (**c**) S3 (with respective slopes of 4.4%, 10% and 15.2%). The crosses and dotted trend line represents the positions of the vortex centers calculated by modeling. (**d**) Interquartile distance between the two populations for the considered devices.

Depending on the spiral design, particles reach equilibrium positions at different locations, it is therefore of interest to analyze the influence of the various parameters (width, slope, AR) on these equilibrium positions. Although we attempted to examine their effects separately by comparing spirals that differ by a single parameter while keeping the others equal or as similar as possible, these parameters are inherently interrelated (for example, changing the channel width also affects the aspect ratio) and may jointly influence particle behavior. First, to study the impact of the upper wall inclination, we experimentally investigated bead trajectories in S2, S1, and S3 that mainly differ in their slopes: respectively 4.4%, 10%, and 15.2%. Figure 5a–c show the distribution of the intensity of 10 μm (blue) and 15 μm (red) fluorescent beads across the width of the channel in these three devices.

For all flow rates, the 10 μm beads formed a well-focused single stream, shifting from an inner position at low flow rates to an outer position as the flow rate increases. The 15 μm beads followed the same behavior. Regarding the impact of the slope on bead positions, lower values yielded initial equilibrium positions closer to the inner wall. This observation is consistent with previous vortex simulation studies: steeper slopes drive vortex centers toward the outer side of the channel even at low flow rates, resulting in an internal equilibrium position farther from the inner wall than in devices with a shallower slope.

Figure 5.d reports the interquartile distance at different flow rates. Here the interquartile distance refers to the gap between the interquartile ranges of the two populations, meaning the interval over which more than 75% of each population are separated. The S3 device, which has the steepest upper wall slope (15.2%), provides an optimal separation (interquartile distance$\:\:\sim\:$10.5 μm) at lower flow rates than the other devices. In line with the more central positions mentioned earlier, this can be explained by the fact that beads that reach a more central initial equilibrium position require less flow velocity, and therefore a lower flow rate, than in other devices to move toward the outer vortices. For S2 and S1 (slopes of 4% and 10% respectively), separation takes place at similar flow rates, between 40 and 60 mL/h, and the separation distance is slightly larger for the S1 device (interquartile distance$\:\:\sim\:$18.4 μm), making it the best suited to the requirements.

**Fig. 6:**
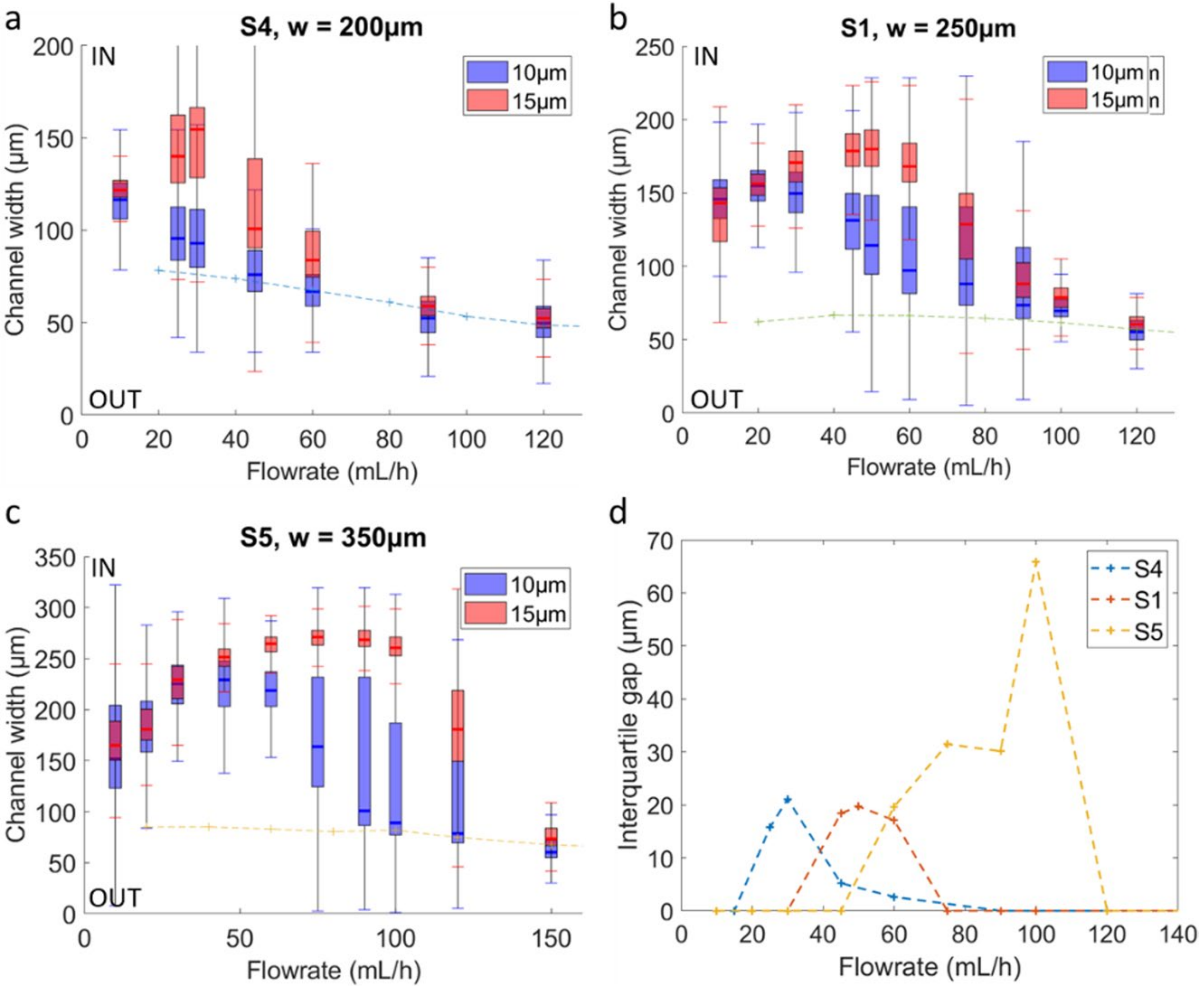
Fluorescence intensity position of 10 μm and 15 μm beads in the normalized channel width at the spiral outlet in (**a**) S4, (**b**) S1 and (**c**) S5 (with respective width of 200 μm, 250 μm and 300 μm). The crosses and dotted trend line represent the positions of the vortex centers calculated by modeling. (**d**) Interquartile distance between the two populations for the considered devices.

The impact of the channel width on the size separation was also investigated. To this end, we compared spirals S4, S1, and S5, which are 200 μm, 250 μm, and 350 μm wide, respectively. In the narrowest channel considered (S4, $\:w=200\:\mu\:m$, Fig. 6a), the vortex centers are located closer to the channel center compared to wider devices (see Supplementary Fig. 4a). One might therefore expect the beads to also focus closer the inner wall. However, even at low flowrates, 10 μm beads are located in the central part of the channel, and from 45 mL/h, their median position closely matches the simulated vortex centers, in the outer part of the channel. This suggests that, due to the narrowness of the channel in this geometry, the vortices are too close to the inner region, preventing the beads from forming a stable equilibrium there, and could lead them to be rapidly drawn into the vortex centers instead. Moreover, it should be noted that the height of the S4 channel is slightly different (75 μm for S4, compared to 73 μm for S1 and S5), which may also contribute, together with the width difference, to the observed effects.

As the channel width increases, the beads tend to stabilize closer to the inner wall, requiring higher flow rates to trigger the outward migration of both 10 and 15 μm beads (Fig. 6b,c). More inward equilibrium positions lead to better separation of the two bead populations (Fig. 6d). This separation, however, occurs at higher flow rates: around 30, 50, and 100 mL/h for channel widths of 200, 250, and 350 μm, respectively. At a fixed flow rate, wider channels exhibit a lower average fluid velocity and increased radius of curvature, which both decrease the Dean number. Consequently, larger channels require higher flow rates to match Dean conditions, pushing separation to higher throughputs. As a result, S5 provides the largest separation distance, but only at higher flowrates, which is contrary to the objective of low flowrate operation. In contrast, although S4 achieves separation similar to that of S1 at lower flowrates (25–30 mL/h), this separation is strongly dependent on flowrate. For S1, the separation remains stable over a wider flow range (45–60 mL/h), ensuring efficient sorting even under flow rate variations and greater versatility when the flow needs to be adjusted to optimize a downstream function.

It should be noted that in devices S5 and S6 (see Supplementary Fig. 5), both of which have a low aspect ratio (~ 0.2), the shift of particles from the inner to the outer equilibrium positions occurs at higher flow rates. This makes these low aspect ratio devices less suitable for reduced flow separation.

Comparisons of separation performance across devices showed that, in a trapezoidal channel, the slope of the upper wall influences the equilibrium position at low flow rates. A steep slope triggers particle separation at a lower flow rate but does not generate sufficiently distinct equilibrium positions for effective separation, unlike a gentler slope. A 10% slope appears to be a good compromise. Additionally, reducing the aspect ratio requires higher flow rates to push particles toward the outer wall and achieve separation, which prevents separation at reduced flow rates. Furthermore, increasing the channel width increases the distance between the focusing positions of the two particle populations but also requires higher flow rates to separate the two populations. Considering these constraints on slope, aspect ratio and width, the S1 device geometry ( $\:w\:=\:250\:\mu\:m,\:h\:=\:73\:\mu\:m,\:\:slope\:=\:10\:\%,\:\:h/w=\:0.29$ ) provided the best separation at moderate flow rates (40–60 mL/h) and was therefore selected for cell isolation.

### Cell separation

Ultimately, these devices are designed to separate cells based on their size. One important application of such size-based separation is the isolation of Circulating Tumor Cells (CTCs), which have emerged as key biomarkers for cancer diagnosis through liquid biopsy, as well as for personalized medicine and treatment monitoring^45,46^. A major challenge in this context lies in isolating the rare CTCs from the large population of blood cells. CTCs typically exhibit diameters ranging from 12 to 20 μm^47^ which partially overlap with those of the blood immune cells. The White Blood Cells (WBCs) population is heterogeneous and mainly consists of neutrophils (approximately 60%, 9–15 μm in diameter) and lymphocytes (around 30%), which include both small (7–8 μm) and large (12–15 μm) subpopulations. Monocytes, although less abundant (~ 5%), are larger, with diameters ranging from 12 to 20 μm^48^. In addition, the isolation of CTCs is further complicated by their extreme rarity, with typically only a few CTCs present among millions of WBCs per milliliter of blood^1^. To evaluate the performance of our spiral devices for CTC isolation, the position at the output of the device S1 of various types of cancer cell lines was investigated: MDA-MB-231 breast cancer cells, MCF7 breast cancer cells, HCC827 lung cancer cells and PC3 prostate cancer cells. Their average diameters are 16.3 (± 2.6), 17.8 (± 3.2), 18.7 (± 3.5), and 19.3 (± 2.7), µm, respectively, consistently with values reported in the literature^49–51^. These cell lines are widely reported as relevant mimicking CTCs^52^, so their behavior in the device S1 was compared to the one of WBC.

**Fig. 7:**
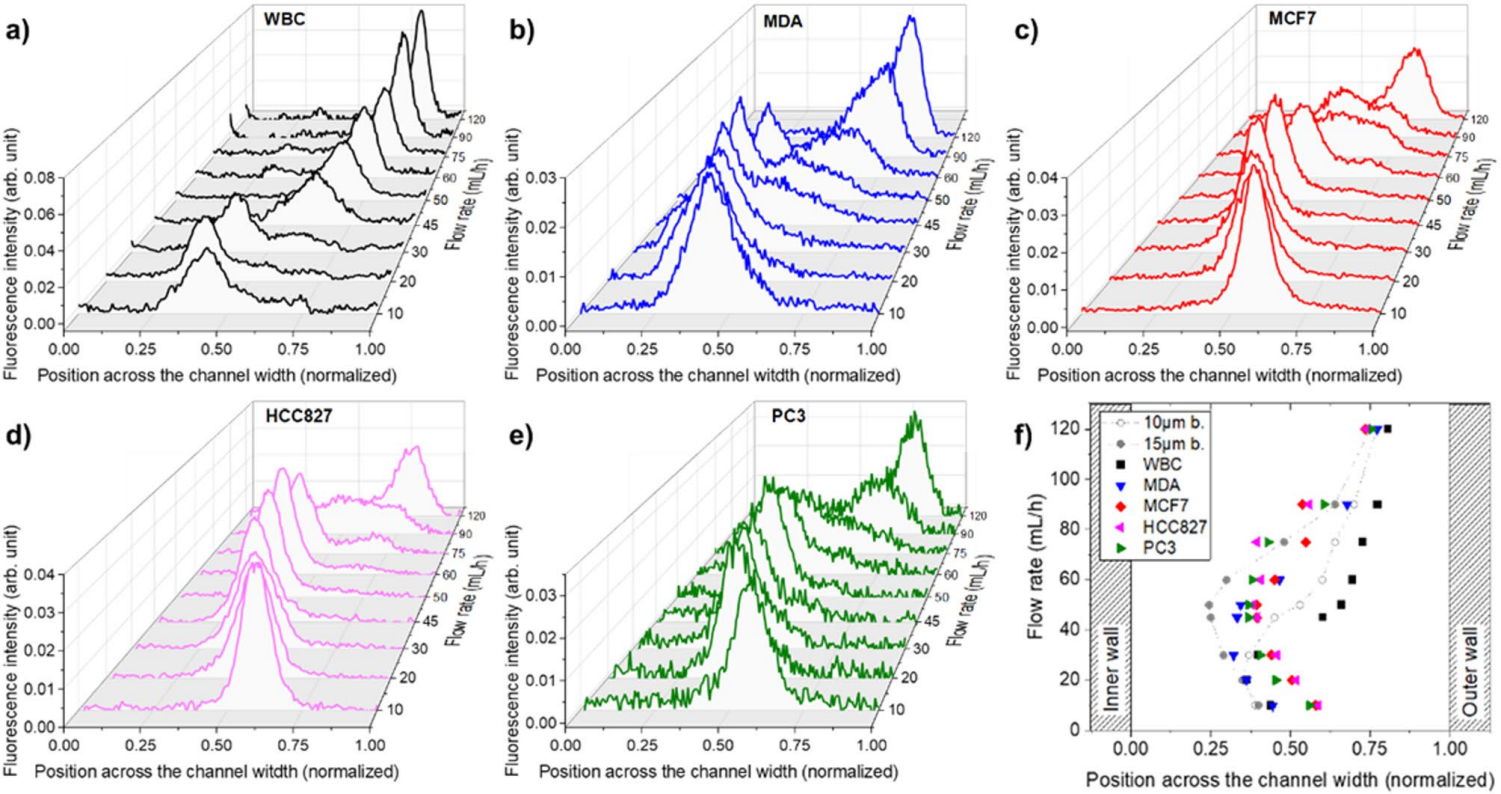
(**a**–**e**) Cells intensity position at the device’s outlet, as a function of the flow rate for (**a**) MDA-MB-231, (**b**) MCF7, (**c**) HCC827, (**d**) PC3 and (**e**) WBCs. (**f**) Median position of different cell type population based on their fluorescence intensity of the channel width at the outlet.

Figure 7.*a-e* reports the positions at the channel outlet of WBCs, MDA-MB-231, MCF7, HCC827 and PC3 according to flow rate. Despite their deformability and higher polydispersity, the cells exhibit similar focusing behavior to that of beads: at low flow rates, they occupy inner equilibrium positions that shift first inwards and then outward as the flow rate increases. The inner position remains farther from the inner wall of the channel than for beads. This is in line with Hur et al.^53^ and Hafemann et al.^36^, that attributes this discrepancy to the larger size heterogeneity and deformability of cells with respect to beads. Size-dependent effects were also observed: larger cells require higher flow rates to reach their inner equilibrium positions and tend to migrate outward at higher flow rates, in agreement with bead-based behavior and previous cited reports (Fig. 7.f). A noticeable difference is found between WBCs and mCTCs, consistent with their size disparity. WBCs (~ 10 μm) exhibit a lateral position shift around 30 mL/h, while mCTCs shift at higher flow rates, starting near 50 mL/h for smaller cells (MDA) and up to 75 mL/h for the largest subtypes (HCC827, PC3).

**Fig. 8:**
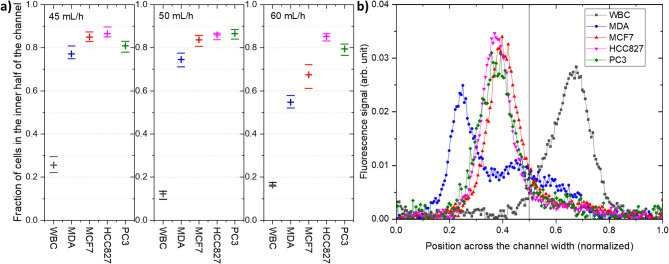
(**a**) Fraction of cells in the inner half of the channel at the exit, for each population, at 45 mL/h (left), 50 mL/h (middle), and 60 mL/h (right). The plus sign markers correspond to the integral of the fluorescence signal intensity over the inner half of the channel section. The horizontal line markers show the uncertainty range owing to the finite size of the cells, thus the lateral extension of the fluorescence signal of single cell. (**b**) Fluorescence signal across the channel width near the outlet, at 50 mL/h, for each cell population. The signal is normalized by the integral of the signal intensity over the channel width.

Based on the fluorescent profiles and considering that the two outlets are separated at the channel midpoint, we determined the proportion of each cell population directed toward the inner or the outer outlets. Figure 8a show the fraction of cells located in the inner part of the channel, calculated from the fluorescence signal intensity, which corresponds to the recovered cells in the inner outlet^54^. The results are shown for each population, at 45 mL/h, 50mL/h and 60 mL/h. Accounting for the fact that the fluorescence signal of a single cell extends over approximately 5% of the channel width (for cells with an average diameter of 15 μm), we introduced a corresponding uncertainty in the position of the separation between the two outlets. At 50 mL/h, WBCs are more effectively removed (89% eliminated, with 11% remaining in the inner outlet), while tumor cell recovery ranges from 75% to 86%, depending on the cell type.

Figure 8b shows the fluorescent signal profile of each cell population across the channel width at a flow rate of 50 mL/h. At this flow rate, the peaks are narrower and the overlap between the signals of cancer cells and white blood cells is indeed reduced (See supplementary Fig. 6). This operating condition is consistent with the low target flow rates discussed previously. To assess the viability of HCC827 cells after passing through the spiral device at flow rates of 30, 60, and 120 mL/h. While the viability of cells in the input suspension was 99%, an average viability of 98% was measured at the outlet, with no observable correlation with the imposed flow rate. These results suggest that cell viability is preserved following passage through the spiral channel.

Other spiral systems reported in the literature that reach recovery rates close to 90% operate at significantly higher flow rates. For instance, Omrani et al.^51^ device operated at 102 mL/h and obtained a removal of 92% of WBC and a recovery rate of 91% of CTC, or Zhu et al.^23^ obtained 94% removal of WBC, and a CTC recovery rate of 97% at 210 mL/h. For CTC isolation and characterization, one would require even higher removal rate, greater than 99.99%. Therefore, beyond single-step approaches, two-step sorting strategies that combine size-based separation with biology-based separation have been developed, demonstrating high isolation performance^12,55^. However, they are currently performed in separated devices operating at different flow rates. By providing effective pre-enrichment at low flow rates, our spiral device could bridge this gap and enable the seamless integration of size-based and biology-based separations within a single workflow.

## Conclusion

In this work, we focused on separating cells according to their size using a passive hydrodynamic method by means of a spiral channel, inducing Dean vortices, at reduce flow rate. Although this method has been used in many studies, understanding of the phenomena involved remains partial, based only on empirical formulas and limited to specific operating conditions. We studied the change in equilibrium position in the channel, from the inner wall to the outer wall of the spiral, at high flow rate. Both experimental work and simulations supported the hypothesis that as the flow rate increases, the particle beams concentrate towards the center of the vortices. These results improve our understanding of particle trajectories and provide guidelines for the design and optimization of future spiral devices.

We sought to design a channel capable of separating cancer cells from white blood cells at low flow rates. Operating at low flow rates increases the versatility of this technique and enables its integration into complex workflows. It also reduces shear stress on cells, making it more suitable for biological applications. In order to optimize the geometry of the channel dedicated to low-flow cell separation, we first compared the trajectories of populations of 10 μm and 15 μm beads along seven different devices with varying geometric parameters such as trapezoidal cross-section, channel width and aspect ratio, as well as their interaction with fluid dynamics. These results highlight clear trends between the geometry of the system, the flow rate and the behavior of biological objects, as well as the main trade-offs to be considered. We selected the most promising device, with the following characteristics: width of 250 μm, upper wall slope of its trapezoidal section of 10% and aspect ratio of 0.29. This device was tested with four different types of cancer cells (MDA-MB-231, MCF7, HCC827, PC3) and white blood cells. The results showed that although these were polydisperse populations, they concentrated well in a beam which, like beads, shifted from the inside to the outside of the channel as the flow rate increased.

At 50 ml/h, and based on the fluorescence signal integrated across the entire width of the channel at the outlet, the most efficient device concentrated 89% of the WBCs in the outer half of the channel at the outlet and between 75% and 86% of the cancer cells, depending on the cell line, in the inner half of the outlet, despite the high heterogeneity of all cell populations. These results reinforce the potential and versatility of Dean’s Vortex and demonstrate the strong potential of this device as a pre-enrichment function in a workflow involving multiple sorting steps.

## Supplementary Information

Below is the link to the electronic supplementary material.

Supplementary Material 1

Supplementary Material 2

## Data Availability

The data supporting the findings of this study are available within the article and its Supplementary Information.
