# Supplementary material for: Effective dean vortex separation at reduced flow rates towards rare cell sorting
Source: Sci Rep. 2026 Feb 25;16:10422. doi: 10.1038/s41598-026-40845-4 (PMC13031336; doi:10.1038/s41598-026-40845-4)
Supplement: Supplementary file 1 — Supplementary Material 1 [file 41598_2026_40845_MOESM1_ESM.pdf]

# Supplementary Information

To evaluate the performance of the single focusing strategy, which consists of focusing a single population while allowing the smallest diameter population to circulate within the vortices, we designed a spiral channel (S0) in which the 15 µm beads have to focus into a single stream, while the 10 µm beads remain unfocused. To achieve this, the height and width of the channel were chosen to satisfy the confinement ratio $\frac{a}{D_\mathrm{h}}$ (where a is the particle diameter, and $D_\mathrm{h}$ is the hydraulic diameter of the channel) and the lift-to-Dean force ratio $R_\mathrm{f}$ for the 15 µm population (Supplementary Table 1), while these conditions were not met for the 10 µm population ($\frac{a_\mathrm{p}}{D_\mathrm{h}} = 0.06 < 0.07$).

| | **10µm beads** | | **15µm beads** | |
|---|---|---|---|---|
| **Spiral** | $\boldsymbol{a/D_\mathrm{h}}$ | $\boldsymbol{R_\mathrm{f}}$ | $\boldsymbol{a/D_\mathrm{h}}$ | $\boldsymbol{R_\mathrm{f}}$ |
| **S0** | 0.063 | 0.049 | 0.094 | 0.110 |

*Supplementary Table 1: Confinement ratio and Lift-to-Dean ratio for the S0 spirals, calculated either below or above their respective focusing thresholds for 15 µm and 10 µm particles, respectively.*

10 µm and 15µm beads were injected in the channel and their fluorescence intensity have been measured in the channel outlet width. The distribution of fluorescent intensity for the two populations is shown in Supplementary Figure a) b) and c) at respectively 30, 60 and 90 mL/h. For all these flow rates, the fluorescence profile of the 10 µm beads in Device S0 appears larger compared to the sharp peaks of the 15 µm beads fluorescence. This indicates that the 10 µm beads are unfocused and can flow through the vortices, as predicted from the design criteria.

Moreover, the central positions of the two bead populations are very close to one another. On Supplementary Supplementary Figure 1.d is represented the proportion of the channel width in which the fluorescence intensity of the two bead populations is greater than 20% of their maximum intensity, for different flow rates. At all tested flow rates, the peaks of the two populations significantly overlap, making separation impossible between 20 mL/h and 120mL/h.

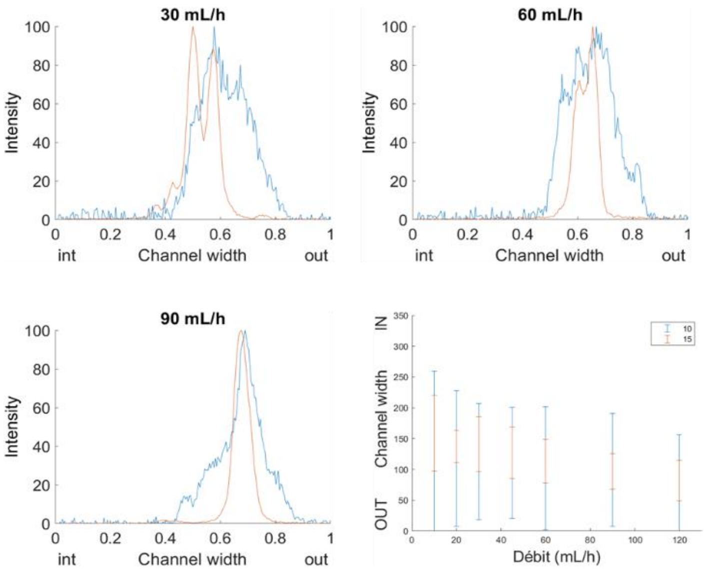


*Supplementary Figure 1: Intensity repartition of 10µm (blue) and 15µm (orange) diameter beads throughout the channel width at the outlet of S0 device at a)30, b) 60 and c) 90 mL/h. d) Proportion of the channel width in which the fluorescence intensity of the two bead populations is greater than 20% of their maximum intensity, for different flow rates.*

| a (µm) | Ratio | S0 | S1 | S2 | S3 | S4 | S5 | S6 | S7 | S8 | S9 |
|---|---|---|---|---|---|---|---|---|---|---|---|
| **10** | $\boldsymbol{a/D_{\mathrm{h}}}$ | 0.063 | 0.103 | 0.094 | 0.120 | 0.102 | 0.098 | 0.130 | 0.092 | 0.079 | 0.073 |
| | $\boldsymbol{R_{\mathrm{f}}}$ | 0.049 | 0.221 | 0.164 | 0.346 | 0.212 | 0.186 | 0.443 | 0.155 | 0.098 | 0.077 |
| **15** | $\boldsymbol{a/D_{\mathrm{h}}}$ | 0.094 | 0.155 | 0.140 | 0.180 | 0.153 | 0.146 | 0.196 | 0.138 | 0.118 | 0.109 |
| | $\boldsymbol{R_{\mathrm{f}}}$ | 0.110 | 0.497 | 0.368 | 0.778 | 0.477 | 0.419 | 0.997 | 0.349 | 0.220 | 0.172 |

*Supplementary Table 2: Confinement ratio $a/D_h$ and ratio of the lift to Dean forces ($R_f$), calculated for tested spirals S0 to S1, for particles of 10 and 15 µm. a is the particle diameter, and $D_h$ is the hydraulic diameter of the channel)*

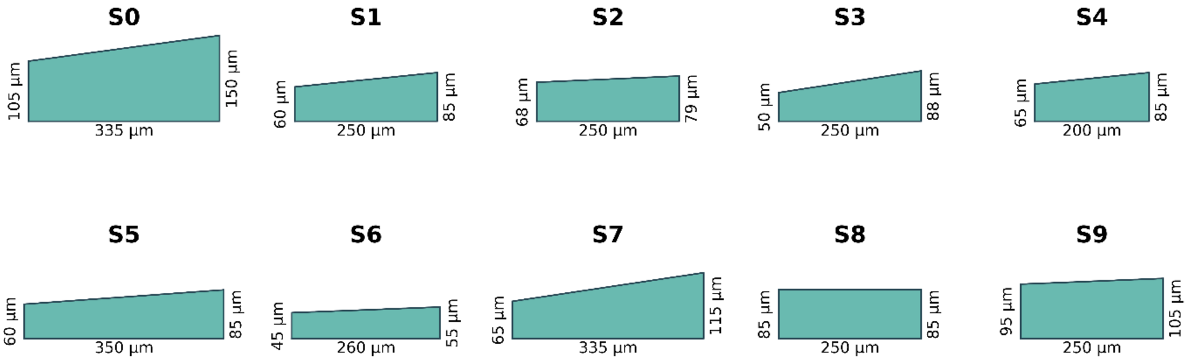


*Supplementary Figure 2: Schematic representation of the spiral sections, presented at a uniform scale.*

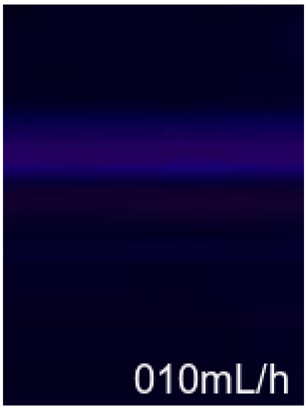


*Supplementary Video 1: Compiled fluorescent images of of 10 and 15 µm diameter beads (in blue and red respectively) at different flowrate showing the inner to outer shift of position of the particles as the flowrate increases. (See attatched file)*

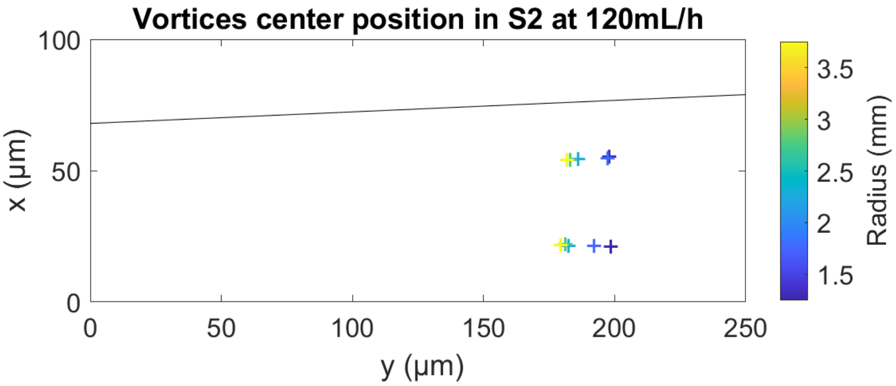


*Supplementary Figure 3: Vortices center positions in S2 cross section at 120 mL/h at the outlet, depending on the radius of curvature. The lowest radius (1.25 mm, blue) corresponds to the most internal spire; and the bigger one (3.75 mm, yellow) corresponds to the last one, at the outlet. x and y axis represent respectively the width and height of the channel section.*

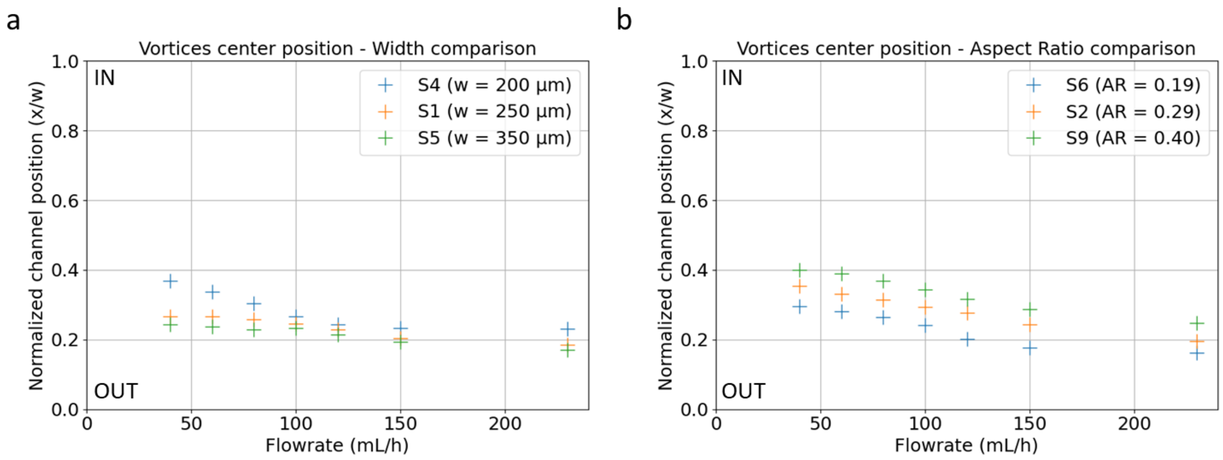


*Supplementary Figure 4: Lateral position of vortex centers across the normalized channel width for varying flow rates. Effect of parameters of interest on vortex center location: a) channel width (w) in S4, S1 and S5. b) aspect ratio (AR) S6, S2 and S9.*

The aspect ratio plays a crucial role in particle size-based positioning. The device S6 has the same width and slope as S2 but differs in height, resulting in a lower aspect ratio. This difference significantly affects the bead positions as shown in Supplementary Figure 5. In S6, the shift toward the outer position occurs at much higher flow rates than in lower-aspect-ratio channels. Moreover, in spiral S6, although both particle populations migrate closer to the inner wall, their streams remain too close to each other for effective size-based separation. At the flow rates of interest (around 50 mL/h), the larger aspect ratio of S6 appears to promote better separation.

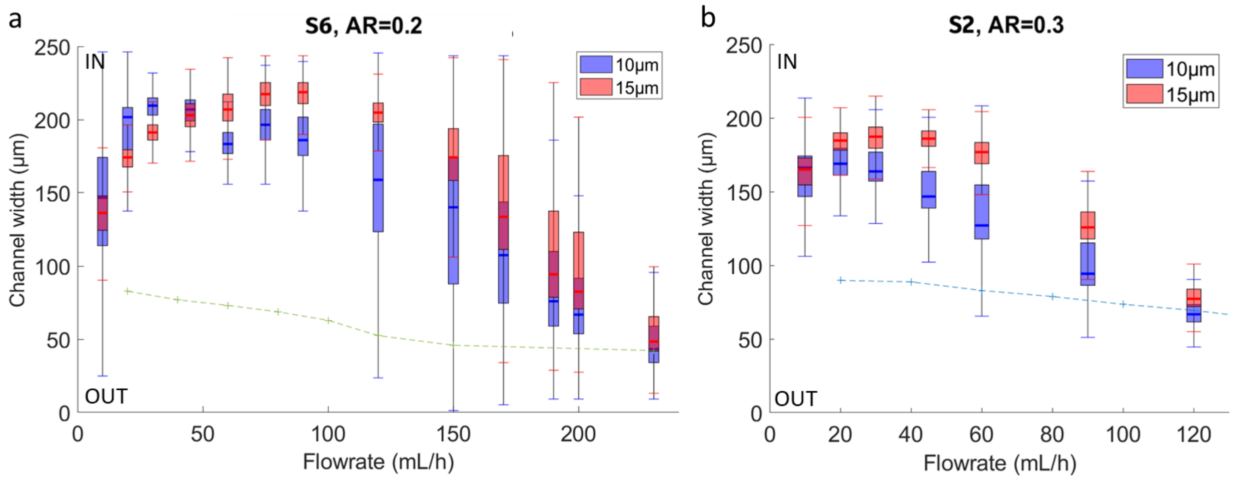


*Supplementary Figure 5: Fluorescence intensity position of 10 µm and 15 µm beads in the channel width at the spiral outlet in a) S6 and b) S2 (with respective aspect ratio (AR) of 0.2 and 0.3).*

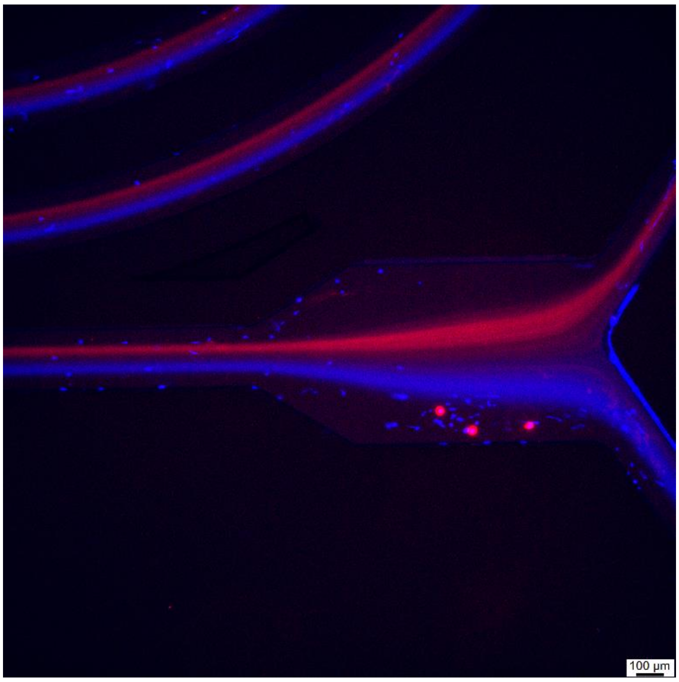


*Supplementary Figure 6: Superimposed fluorescence image of WBC in blue ($10^6$ cell/mL, stained with Hoechst) and MCF7 in red ($10^3$ cell/mL, stained with red CellTracker, Invitrogen) flowing through the outlet of the channel.*
