# Supplementary figures and images for: Effective dean vortex separation at reduced flow rates towards rare cell sorting
Source: Sci Rep. 2026 Feb 25;16:10422. doi: 10.1038/s41598-026-40845-4 (PMC13031336; doi:10.1038/s41598-026-40845-4)

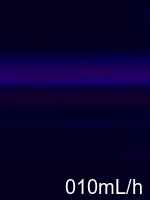

Supplement: Supplementary file 2 — Supplementary Material 2 [file 41598_2026_40845_MOESM2_ESM.gif]
